# Integrated analysis of transcriptome and metabolome reveals the molecular interactions and regulation of muscle flavor precursors in Tengchong Snow chickens and AA broilers

**DOI:** 10.3389/fvets.2026.1760840

**Published:** 2026-03-10

**Authors:** Min Yang, Ruifang Zhang, Jingying Zhao, Zonghui Jian, Hao Wu, Xiannian Zi, Kun Wang, Zhiqiang Xu, Changrong Ge, Junjing Jia, Lixian Liu, Tengfei Dou

**Affiliations:** 1 Faculty of Animal Science and Technology, Yunnan Agricultural University, Kunming, China; 2 Institute of Science and Technology, Chuxiong Normal University, Chuxiong, China; 3 Faculty of Animal Husbandry and Veterinary Medicine, Yunnan Vocational and Technical College of Agriculture, Kunming, China; 4 College of Food Science and Technology, Yunnan Agricultural University, Kunming, China

**Keywords:** flavor precursors, integrated analysis, metabolomics, Tengchong Snow chicken, transcriptomics

## Abstract

The flavor of chicken meat is formed by a series of complex chemical reactions, and the flavor precursors are affected by regulatory genes. In order to study the differences of muscle flavor precursors between Tengchong Snow chickens and AA broilers, integrated metabolomics and transcriptomics analyses were used to investigate muscle metabolite profiles and the key genes involved in the metabolism of muscle flavor compounds. The results showed that 42 significantly differentially metabolites were detected, and (5-L-Glutamyl)-L-glutamate, gamma-Glutamylalanine, S-Adenosylhomocysteine, Homo-L-arginine and GMP were important flavor metabolites. The key candidate genes with high correlation with flavor precursor metabolites were identified through correlation analysis as *NME2*, *AMPD1*, *GADL1*, *ASNS*, *CARNS1*, and *CTH*. In addition, the gene-metabolite interaction network for flavor formation in chicken breast muscle was constructed. This study could provide some basic data for the formation mechanism of local chicken excellent meat quality, and provide reference for the development and utilization of local chicken breeds and the selection and breeding of high-quality broilers.

## 1 Introduction

With the improvement of human living standards and the continuous dietary restructuring, the demand for meat products has gradually undergone fundamental changes from quantity to quality, and higher requirements have been put forward for sensory satisfaction, flavor and nutritional value. As a result, chicken quality and nutrition have become public concerns, with flavor standing out as a key indicator of meat quality (1). Moving forward, a major challenge for the poultry industry will be enhancing chicken meat quality without compromising yield. Therefore, in order to improve the quality of chicken meat and improve its meat flavor, a growing number of researchers study the evaluation method of chicken flavor and nutritional regulation.

The flavor of chicken is affected by many factors, including the variety, age, diet, feeding method, post-slaughter curing, processing and storage, and cooking method, etc. These factors will affect the composition and content of chicken flavor precursors, or affect the process of flavor formation reaction during cooking. The main flavor precursors found in meat can be divided into two categories: water-soluble components and lipids. Water-soluble components include free sugars, nucleotide-bound sugars, free amino acids, peptides, nucleotides, and other nitrogen-containing compounds. Chicken’s umami flavor comes mainly from water-soluble precursors such as inosine acid (IMP) and glutamate (2, 3). IMP or ribose reacts with a sulfur-containing amino acid (cysteine or cystine) during cooking to form 2-methyl-3-furanthiol, an important flavor compound that produces the meat flavor of chicken broth (4). Lipids refer to fat and fat-soluble substances in meat. Intramuscular fat contains phospholipids that carry a large amount of unsaturated fatty acids and are rich in oleic acid, linolenic acid and arachidonic acid (ARA). A high percentage of ARA has been proved to help chicken obtain better sensory properties (5, 6). The main component of intermuscular fat and subcutaneous fat is triglyceride, which has little effect on flavor. However, the key metabolites affecting chicken flavor and the molecular mechanism of flavor formation have not been fully elucidated, and the flavor precursors, characteristic flavor compounds and their production mechanisms need to be further studied and determined.

Metabolomics could identify, screen and label different metabolites related to meat quality, which is helpful to understand the correlation between meat quality and variety, age and storage time. Some study used metabolomics and multivariate statistics to detect creatine, carnosine, amino acids and nucleotides in breast muscle and leg muscle of Camellia chicken, which could be used as biomarkers of taste contribution substances in different parts of Camellia chicken (7). Changes in gene expression will be affected by changes in metabolic processes, and analysis of metabolome could reveal its biology and molecular mechanism. Combined analysis of metabolome and other sequencing techniques could explore the molecular regulatory mechanisms in the formation of meat flavor (8). Transcriptome sequencing technology can further analyze the regulatory factors related to these phenotypic traits, and screen and identify the genes related to meat quality and flavor traits at the molecular level. Some study conducted transcriptome sequencing on the breast muscle and leg muscle tissues of 42-day-old and 90-day-old Peking You chickens, and the results showed that *PPARG*, *LPL*, *FABP4*, *THRSP*, *RBP7*, *PLIN* and *LDLR* genes had important regulatory effects on intramuscular fat deposition (9). But transcriptomic sequencing can only provide information at the gene level, cannot investigate the true level of an organism’s metabolism, and is difficult to identify key pathways responsible for regulating specific traits. Combined analysis of transcriptome and metabolome could accurately explore the key regulatory metabolic pathways and regulatory genes in the formation of flavor, and study the molecular regulatory mechanisms in the formation of meat flavor (8).

Currently, the commercial broiler chickens such as AA broilers and Kobe broilers are struggling to gain a larger market share, due to its excellent growth performance, high feed conversion rate, and uniform carcass traits, it has become the most widely used commercial breed in the world for broiler production. Especially, AA broiler is one of the earliest white-feathered fast-growing broiler breeds introduced into China, with a longer history of local adaptation, more stable stress resistance, and high market share, making it a commonly used reference in scientific research comparing with local chicken breeds. However, the meat quality of the commercial broiler chickens is poor. Tengchong Snow chickens is a local chicken breed with black skin, muscles and bone in Yunnan Province of China. It shows the excellent meat quality for containing abundant flavor substances in its muscles, and the meat is firm and delicious, which meeting the high-quality consumption demands, but the formation mechanism of meat flavor is still unclear. In this study, Tengchong Snow chickens and AA broilers were selected as the research subject and the control group respectively, and the differences of muscle flavor precursors between Tengchong Snow chickens and AA broilers were studied by combining metabolomics and transcriptomics to provide basic data for the formation mechanism of good meat quality in local chickens.

## 2 Materials and methods

### 2.1 Animals and sample collection

The experiment was carried out in the experimental chicken Farm of Yunnan Agricultural University. 30 Tengchong Snow chickens (TC group) and 30 AA broilers (AA group) were raised under the same dietary nutrition level and feeding management level. Feeding to market age (AA broilers at 42 days; Tengchong Snow chickens at 150 days) were slaughtered and sampled.

After feeding to market age, 30 chickens were selected for slaughter, and the feeding was cut off 12 h before slaughter. Six breast muscle samples (100 mg) were collected from each chicken and placed into RNase-free tubes, immediately frozen in liquid nitrogen, and then stored at −80 °C for subsequent transcriptome, metabolome and Real-time PCR gene expression analysis.

### 2.2 Metabolomic analysis of muscle tissue

Metabolomic analysis was conducted on breast muscle samples from 6 Tengchong Snow chickens and 6 AA broilers. 800 μL precooled methanol/acetonitrile (1:1, v/v) was added to the homogenized sample for cyclone. The sample was ultrasonically treated in a cold water bath, and then placed at-20 °C for 1 h. The mixture was centrifuged for 15 min (13,000 rpm, 4 °C), the supernatant was collected and freeze-dried. UHPLC (1,290 infinity LC, Agilent Technologies), HILIC and RPLC were used for sample separation. The column temperature was 25 °C, the flow rate was 300 μL/ min, and each sample was loaded with 2 μL. Chromatographic mobile phase consisted of buffer A (water +25 mM ammonium acetate +25 mM ammonium hydroxide) and buffer B (acetonitrile). The gradient was 95% buffer B for 1 min, linearly decreased to 65% within 13 min, and then decreased to 40% after 2 min. Keep it at 40% for 2 min, then 0.1 min to 95%, finally 95% for 5 min. In order to avoid the influence of signal function, the samples were randomly analyzed.

Sample analysis was performed using UHPLC coupled with a quadrupole time-of-flight mass spectrometer (AB SCIEX TripleTOF 5,600) in ESI positive and negative modes. ESI source conditions after HILIC separation were as follows: ion source Gas1 (Gas1) was 60, ion source Gas2 (Gas2) was 60, curtain gas was 30, source temperature was 600 °C, ion spray voltage floats were 5,500 V in positive mode and −5,500 V in negative mode. In the mass spectrum acquisition, the instrument was set to m/z range as 60–1,000 Da, product ion scan m/z range was 25–1,000 Da, TOF mass spectrometry scan accumulation time was 0.20 s/spectrum, product ion scan accumulation time 0.05 s/spectrum. Product ion scanning uses information dependent acquisition (IDA) and selects high sensitivity mode. The collision energy was fixed at 35 V ± 15 eV. The cluster potential was set to ± 60 V. IDA Settings were as follows: Exclude isotopes within 4 Da, Candidate ions to monitor per cycle: 6.

The MSConvert tool in Proteowizard package (v3.0.8789) (10) converts the original mass spectrometry file to mzXML file format. The quantitative list of substances was obtained by using R XCMS software package for peak detection, peak filtering and peak alignment. Public databases HMDB, massbank, LipidMaps, mzcloud, KEGG and self-built material bank were used to identify the substances. Significant differential metabolites (SDMs) were screened using R software package Ropl (11), and the screening criteria were *p*-value < 0.05 and Variable Importance in the Projection (VIP) ≥ 1. The sample data were analyzed by Principal Component Analysis (PCA) and Orthogonal Partial Least Squares Discriminant Analysis (OPLS-DA). MetPA data base^1^ was used to perform functional pathway enrichment analysis of SDMs.

### 2.3 Transcriptomic analysis of muscle tissue

Transcriptome analysis was conducted using the same breast muscle tissue samples (6 samples per group) as in the metabolomics analysis. Total RNA of breast muscle samples was isolated using TaKaRa MiniREST universal RNA extraction kit (TaKaRa Biotechnology Co., Ltd., Dalian, P. R. China) according to the manufacturer’s protocol. Purity was measured by a Nanodrop 2000 spectrophotometer (Nanodrop, Wilmington, DE, United States). The OD260/OD280 ratio of all samples ranged from 1.8 to 2.0. Total RNA concentration was determined using Qubit 2.0 fluorimeter (Termo Fisher scientific Inc., MA, United States) and integrity was detected using Angilent 2,100 bioanalyzer (Agilent Technologies, Inc., Santa Clara, CA, United States). The mRNA with polyA structure in total RNA was enriched by Oligo (dT) magnetic beads, and the RNA was broken into fragments of about 300 bp in length by ion interruption. Using RNA as template, 6 base random primers and reverse transcriptase were used to synthesize the first strand cDNA, and the second strand cDNA was synthesized using the first strand cDNA as template. After the library construction was completed, the next generation sequencing (NGS) Illumina Hiseq 4,000 was used to perform paired end (PE) sequencing on these libraries based on the Illumina sequencing platform.

The sample was sequenced on the computer to obtain an image file, which was converted by the sequencing platform’s built-in software to generate raw data for FASTQ. Raw reads obtained by sequencing were filtered and the 3′ end-band junction sequences were removed by Cutadapt. Reads with a mean mass score lower than Q20 were removed, and the error rate and GC content distribution of the sequencing data were examined to obtain clean reads for subsequent analysis. The filtered reads were compared to the reference genome for differential expression analysis using the upgraded HISAT2 software of TopHat2.^2^

Gene expression analysis was used DESeq2^3^ to analyze gene expression. Padj (FDR) represents the significance of differentially expressed genes, and log2FoldChange (log2FC) represents the relative expression level of genes. log2FC ≥ 1 and FDR < 0.05 were used as screening criteria to obtain the final differentially expressed genes (DEGs). KEGG enrichment analysis of DEGs was performed using KOBAS^4^ to explore their possible biological functions (12).

To verify the results of RNA-seq, 7 genes related to meat flavor formation were randomly selected for qRT-PCR. Design gene specific primers using NCBI (Supplementary Table S1). Total RNA was extracted from the breast muscle using the same animals as the transcriptome and metabolome analyses. The relative expression of each gene was calculated by 2^−ΔΔCt^ method, and each sample was repeated three times (13).

### 2.4 Integrated metabolome and transcriptome data association analysis

The DEGs in the KEGG pathway where flavor precursors were enriched were selected. Spearman correlation analysis was used to analyze the correlation coefficient between DEGs and flavor precursors metabolites. The role of Spearman correlation analysis in this study is to conduct a preliminary screening, aiming to identify the candidate gene sets that are most closely associated with the target trait at the genomic level, and providing targets for subsequent in-depth functional validation studies. Heat map of metabolite and gene correlation using Origin.^5^ Cytoscape (v.3.9.0 https://cytoscape.org/) was used to plot the correlation network diagram between metabolites and genes.

## 3 Results

### 3.1 Differential metabolites and related metabolic pathways of breast muscle tissue in different chicken breeds

**Figure 1:**
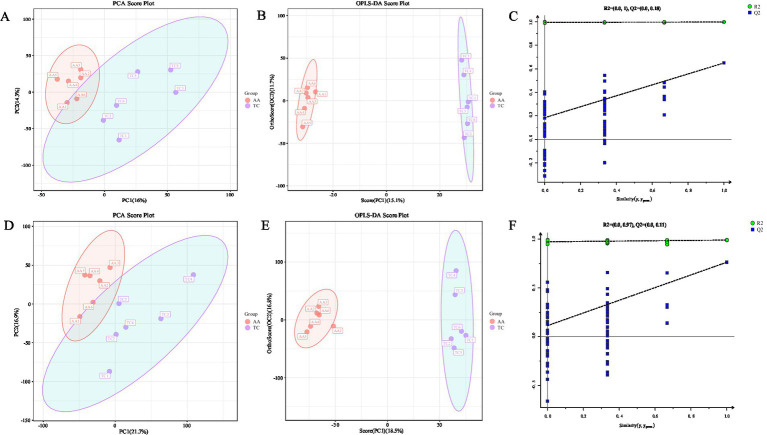
Metabolome quality control analysis. **(A)** PLS-DA score chart of positive ion mode. **(B)** Positive ion mode OPLS-DA score chart. **(C)** Positive ion mode displacement test chart. **(D)** PLS-DA score chart in negative ion mode. **(E)** Negative ion mode OPLS-DA score chart. **(F)** Negative ion mode displacement test chart. In PLS-DA and OPLS-DA plots, the horizontal axis represents the interpretation of the first principal component, while the vertical axis represents the interpretation of the second principal component. The points represent experimental samples, and their colors indicate different groups. The more clustered the samples within a group and the more dispersed between groups, the more reliable the results. For OPLS-DA, the R2 (explaining model variance for X and Y variable datasets) and Q2 (model predictability) score plots can be used to evaluate the classification performance. In the OPLS-DA permutation test plot, if all blue Q2 points are lower than the rightmost original blue Q2 point, the results are considered reliable and valid.

The PLS-DA method was used to analyze the metabolic data of breast muscle tissue from two chicken breeds as a whole. The results showed that the two groups of samples could be clearly distinguished, indicating a significant difference in breast muscle metabolites between TC and AA (Figures 1A,D). In order to further verify the separation of samples of the two chicken species, and screen key metabolites, OPLS-DA analysis was conducted, and the results showed that the samples of the TC group and the AA group were significantly separated, and the data model had a good fit and prediction ability (Figures 1B,E). Model validation was conducted through permutation testing, indicating that the model has good predictive ability and reliability (Figures 1C,F).

**Figure 2:**
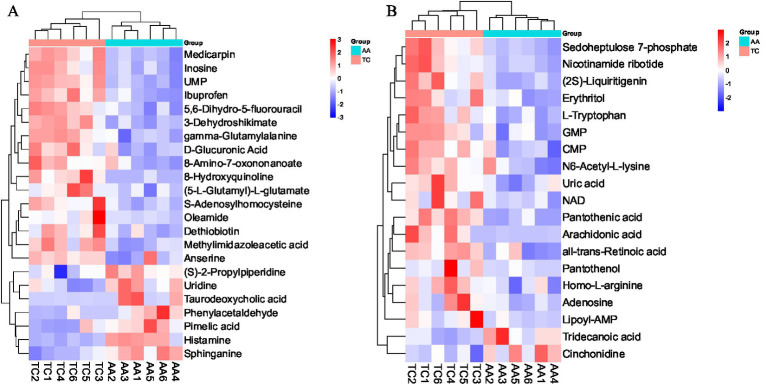
Heat maps of SDMs in positive and negative ion mode of two chicken breeds. **(A)** Heat map of SDMs in positive ion mode. **(B)** Heat maps of SDMs in the negative ion mode.

Compared with AA broilers, 33 SDMs were upregulated and 9 SDMs were downregulated in Tengchong Snow chickens (Table 1). The SDMs could be more intuitively displayed through hierarchical clustering analysis to show the relationships between samples and the differences in the expression patterns of SDMs in different samples (Figure 2). In the positive ion mode, 16 SDMs were upregulated and 7 SDMs were downregulated (Table 1; Figure 2A). In the negative ion mode, 17 SDMs were upregulated and 2 SDMs were downregulated (Table 1; Figure 2B). SDMs from breast muscle tissue of two chicken breeds were enriched by KEGG pathway as shown in Table 2, biotin metabolism, vascular smooth muscle contraction, histidine metabolism, intestinal immune network for IgA production, and purine metabolism pathway were significant enrichment pathways.

**Table 1:** SDMs in Tengchong Snow chickens and AA broilers.

Metabolite	VIP	log2(Fold change)	*P* value	Ion mode
8-Hydroxyquinoline	2.33236	−1.90	0.000046	Positive
Dethiobiotin	1.70382	1.07	0.018189	Positive
S-Adenosylhomocysteine arginine	2.00669	0.51	0.003191	Positive
Medicarpin	2.25994	−1.19	0.000165	Positive
gamma-Glutamylalanine	1.79364	1.08	0.019568	Positive
Inosine	2.17072	0.32	0.000592	Positive
Oleamide	1.68429	1.54	0.032907	Positive
3-Dehydroshikimate	2.29982	−1.30	0.0001	Positive
Ibuprofen	2.2454	−0.69	0.000279	Positive
(5-L-Glutamyl)-L-glutamate	1.58251	1.36	0.032565	Positive
Anserine	1.66692	1.76	0.038993	Positive
D-Glucuronic Acid	1.8366	0.77	0.009322	Positive
UMP	2.37986	−4.72	0.00002	Positive
5,6-Dihydro-5-fluorouracil	2.09405	0.38	0.001354	Positive
Methylimidazoleacetic acid	2.08779	0.42	0.001384	Positive
8-Amino-7-oxononanoate	1.78965	1.03	0.013563	Positive
(S)-2-Propylpiperidine	1.42316	3.08	0.048962	Positive
Sphinganine	2.19638	−0.32	0.000478	Positive
Pimelic acid	1.83596	0.68	0.008851	Positive
Phenylacetaldehyde	1.812	0.90	0.011636	Positive
Uridine	1.60117	2.10	0.042453	Positive
Histamine	2.32369	−1.35	0.000053	Positive
Taurodeoxycholic acid	1.47951	2.56	0.048759	Positive
Arachidonic acid	1.69255	0.61	0.007429	Negative
Pantothenic acid	1.96872	0.47	0.002222	Negative
Lipoyl-AMP	1.50929	1.15	0.028338	Negative
Pantothenol	1.355	1.65	0.032936	Negative
(2S)-Liquiritigenin	1.96646	−0.28	0.000572	Negative
Nicotinamide ribotide	1.83413	0.52	0.003341	Negative
Sedoheptulose 7-phosphate	2.02029	−0.36	0.000474	Negative
L-Tryptophan	1.72671	0.71	0.009268	Negative
GMP	1.37691	2.21	0.047726	Negative
NAD	1.31235	2.32	0.048427	Negative
Uric acid	1.53323	1.04	0.016042	Negative
Adenosine	1.30318	4.25	0.049154	Negative
Erythritol	1.72966	1.03	0.012102	Negative
all-trans-Retinoic acid	1.73913	0.59	0.006183	Negative
N6-Acetyl-L-lysine	1.45197	1.91	0.040802	Negative
Homo-L-arginine	1.36453	1.14	0.028221	Negative
CMP	1.76632	0.60	0.006326	Negative
Cinchonidine	1.53899	1.06	0.017897	Negative
Tridecanoic acid	1.54453	0.87	0.010035	Negative

The SDMs with log2(Fold change) > 0: compared to AA broilers, the SDMs in Tengchong Snow chickens were upregulated. The SDMs with log2(Fold change) < 0: compared to AA broilers, the SDMs in Tengchong Snow chickens were downregulated.

**Table 2:** Enrichment results of KEGG pathway of SDMs.

Pathway id	Pathway name	Compound name	*P* value
gga00780	Biotin metabolism	8-Amino-7-oxononanoate, Dethiobiotin, Pimelic acid	0.005877
gga04270	Vascular smooth muscle contraction	Adenosine, Arachidonic acid	0.018830
gga00340	Histidine metabolism	Histamine, Anserine, Methylimidazoleacetic acid	0.024379
gga04672	Intestinal immune network for IgA production	all-trans-Retinoic acid	0.026738
gga00230	Purine metabolism	GMP, Adenosine, Inosine, Uric acid	0.037292

### 3.2 Differentially expressed genes and associated metabolic pathways of breast muscle tissue in different chicken breeds

As shown in Supplementary Table S2, the samples were sequenced on the computer, and The Raw Data was 153.44GB and 545,847,226 sequences were obtained. After further filtering the sequencing data, 144.5GB and 513,750,414 high quality sequences were obtained. The Q20 quality value was above 97%, and the Q30 quality value of each sample after filtration was above 94%. Compared to the reference *Gallus_gallus* genome, the comparison rate of each sample was higher than 80%. The sequencing data was reliable and can be used for subsequent analysis.

**Figure 3:**
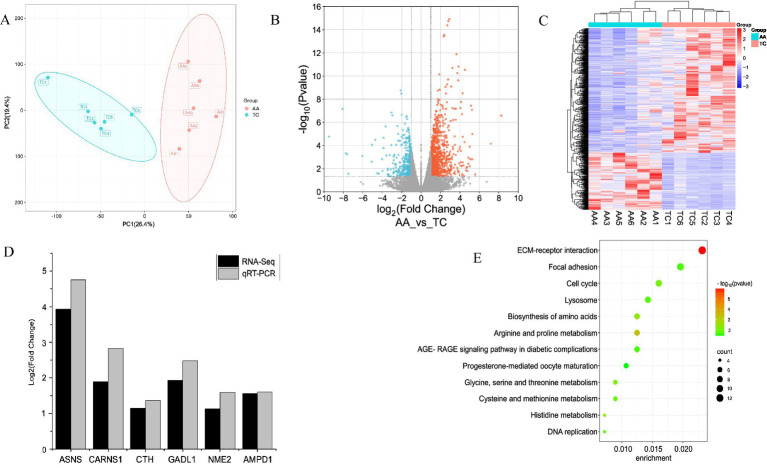
Transcriptome analysis of TC and AA breast muscle tissue. **(A)** PCA plots of TC (blue) and AA (red). **(B)** DEGs heat maps for both chicken species. Red indicates highly expressed genes and blue indicates low-expressed genes. **(C)** DEGs volcano map of two chicken species. Orange dots indicate significantly up-regulated differential genes, blue dots indicate significantly down-regulated differential genes, and gray dots indicate insignificant differential genes. **(D)** qPCR validation of RNA-seq results. **(E)** Bubble diagram of KEGG pathway with significant enrichment of DEGs.

To check the accuracy of the samples, PCA was performed (Figure 3A), and it was found that the two groups of samples were significantly separated. DESeq was used for differential analysis of gene expression, and a total of 15,250 differentially expressed genes were obtained. |log2FoldChange| ≥ 1 and FDR ≤ 0.05 were used as the screening criteria for DEGs. A total of 561 DEGs were identified in the TC vs. AA group. Among them, there were 146 up-regulated genes and 415 down-regulated genes in the TC group (Figures 3B,C; Supplementary Table S3). qRT-PCR verification showed that the expression trend of the 7 selected genes was similar to that of the RNA-seq data (Figure 3D), and the sequencing results were reliable. After KEGG enrichment analysis of 561 DEGs, 12 significantly enriched KEGG pathways were obtained (*p* < 0.05) (Figure 3E; Table 3). Among them, there are 5 pathways related to meat quality, namely arginine and proline metabolism, amino acid biosynthesis, histidine metabolism, glycine, serine and threonine metabolism, and cysteine and methionine metabolism.

**Table 3:** Significantly enriched KEGG pathways.

Pathway id	Pathway name	*P* value	Differentially expressed genes
gga04512	ECM-receptor interaction	0.000001	*COL1A2, COL6A2, RELN, FREM1, COL4A1, COL4A2, THBS4, LAMC1, LAMA4, FREM2, COL6A1, FN1*
gga00330	Arginine and proline metabolism	0.000446	*P4HA3, OAT, CKMT1B, CARNS1, PYCR1, CKB*
gga01230	Biosynthesis of amino acids	0.004563	*PRPS2, ASNS, PGK2, CTH, PYCR1, GAPDH*
gga00340	Histidine metabolism	0.005617	*HNMT, CARNS1, HDC*
gga00260	Glycine, serine and threonine metabolism	0.008522	*AGXT2, SARDH, CTH*
gga04110	Cell cycle	0.010205	*CDK1, MCM5, CDC20, E2F1, PLK1, MCM3*
gga00270	Cysteine and methionine metabolism	0.013902	*AGXT2, LDHA, AHCY, CTH, MAT1A*
gga03030	DNA replication	0.016338	*MCM3, RNASEH2B, MCM2, MCM5*
gga04142	Lysosome	0.020176	*SLC11A1, AP3B2, LAPTM4B, CTSC, GUSB, CTSS, TCIRG1*
gga04510	Focal adhesion	0.022302	*ARHGAP5, COL1A2, COL6A2, RELN, COL4A1, COL4A2, THBS4, LAMC1, LAMA4, COL6A1, FN1*
gga04933	AGE-RAGE signaling pathway in diabetic complications	0.023642	*COL1A2, COL4A1, COL4A2, COL3A1, EGR1, SMAD3*
gga04914	Progesterone-mediated oocyte maturation	0.038341	*CDK1, HSP90AB1, CPEB4, PLK1, CCNB1, HSP90AA1*

### 3.3 Integrated analysis of metabolome and transcriptome

**Figure 4:**
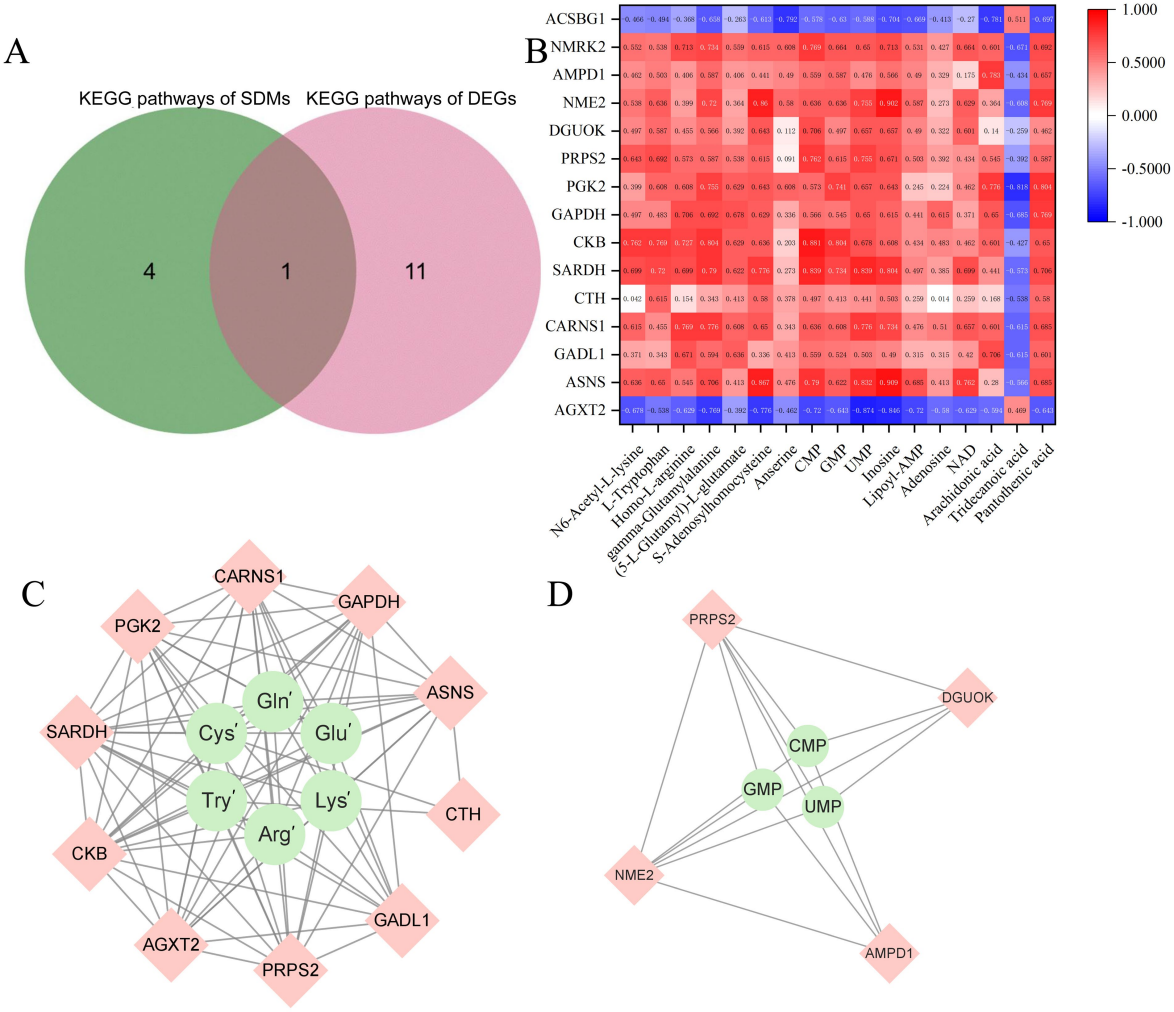
**(A)** Venn diagram of KEGG pathways between DEGs and SDMs. **(B)** Correlation heat maps of selected flavor precursors SDMs and DEGs. Red indicates positive correlation and blue indicates negative correlation. **(C)** Interaction network between amino acids related substances (Glu’, Gln’, Cys’, Tyr’, Lys’, and Arg’ are represented as (5-L-Glutamyl)-L-glutamate, gamma-Glutamylalanine, S-Adenosylhomocysteine, L-Tryptophan, N6-Acetyl-L-lysine, Homo-L-arginine respectively) and DEGs (*PGK2*, *CARNS1*, *GAPDH*, *ASNS*, *CTH*, *GADL1*, *PRPS2*, *AGXT2*, *CKB*, *SARDH*). **(D)** Interaction network between nucleotides (CMP, GMP, UMP) and DEGs (*PRPS2*, *DGUOK*, *AMPD1*, *NEM2*).

There was 1 common KEGG pathway in the metabolome and transcriptome (Figure 4A). Spearman calculations was used to conduct correlation analysis between selected DEGs and selected SDMs. It was showed a strong correlation between DEGs and SDMs (Figure 4B; Supplementary Table S4). (5-L-Glutamyl)-L-glutamate, Adenosine, Anserine, Arachidonic acid, CMP, gamma-Glutamylalanine, GMP, Homo-L-arginine, Inosine, Lipoyl-AMP, L-Tryptophan, N6-Acetyl-L-lysine, NAD, Pantothenic acid, S-Adenosylhomocysteine and UMP showed strong positive correlation with *ASNS*, *GADL1*, *CARNS1*, *CTH*, *SARDH*, *CKB*, *GAPDH*, *PGK2*, *PRPS2*, *DGUOK*, *NME2*, *AMPD1* and *NMRK2*. Specially, (5-L-Glutamyl)-L-glutamate, gamma-Glutamylalanine, S-Adenosylhomocysteine, L-Tryptophan, N6-Acetyl-L-lysine, Homo-L-arginine as for the substances related to glutamate (Glu), glutamine (Gln), cysteine (Cys), tryptophan (Tyr), lysine (Lys), and arginine (Arg) respectively, showed strong positive correlation with *PGK2*, *CARNS1*, *GAPDH*, *ASNS*, *CTH*, *GADL1*, *PRPS2*, *AGXT2*, *CKB*, *SARDH*, and CMP, GMP, UMP showed strong positive correlation with *PRPS2*, *DGUOK*, *AMPD1*, *NEM2* (Figures 4C,D).

## 4 Discussion

Flavor is an important factor affecting the palatability and consumer preference for meat. The flavor profile of meat arises from volatile aroma compounds and non-volatile taste substances generated through heat-induced reactions of precursor compounds. These substances are characterized by their trace amounts, diversity, and complexity, playing a decisive role in the development of meat’s distinctive flavor (14). Tengchong Snow chicken is a local breed of Yunnan Province, is known for its high nutritional value, excellent meat and egg quality, and other desirable germplasm traits. However, the mechanisms underlying its meat flavor formation remain unclear. Flavor precursors serve as the foundation for meat flavor development. In this study, transcriptomics and metabolomics were used to analyze the formation mechanism of muscle flavor precursors and the key gene-metabolites for Tengchong Snow chickens.

Through metabolome analysis, 42 SDMs were identified, among which 16 SDMs were associated with flavor precursor formation, predominantly amino acids and nucleotides. KEGG pathway enrichment analysis revealed significant enrichment in biotin metabolism, histidine metabolism, and purine metabolism, which may be closely linked to flavor development. Biotin, an essential vitamin in animal metabolism, plays a crucial role in blood glucose stability, sugar and lipid metabolism, and is indispensable for initiating fatty acid synthesis and extending the fatty acid carbon chain (15, 16). Histidine is primarily involved in regulating animal growth and enhancing muscle antioxidant capacity and carnosine content (17). Purine metabolism, a key pathway influencing flavor, is closely associated with the generation and degradation of inosine monophosphate (IMP) and adenosine monophosphate (AMP). Amino acids serve as important flavoring substances and precursors of volatile compounds, constituting a significant component of meat flavor (18). The final flavor of meat is not determined by a single amino acid, but by the combination and ratio of many flavored amino acids (19). The degradation of peptides and amino acids in meat further enhances its sensory characteristics and taste (20).

In this study, the abundances of (5-L-Glutamyl)-L-glutamate, gamma-Glutamylalanine, S-Adenosylhomocysteine, L-Tryptophan, N6-Acetyl-L-lysine, Homo-L-arginine in Tengchong Snow chickens were higher than that in AA broilers, and these SDMs are the substances related to glutamate, glutamine, cysteine, tryptophan, lysine, and arginine, respectively. Glutamate and glutamine are both fresh-tasting and functional amino acids known to enhance antioxidant and immune abilities, as well as improve intestinal growth, development, and health (21). Lysine is a sweet amino acid, which plays an important physiological role in the synthesis of proteins and peptides such as muscles, enzymes, serum proteins and hormones in animals (22). Tryptophan and arginine are bitter-tasting amino acids. Tryptophan promotes the animals growth and development, regulates metabolic and physiological functions, and improves the body’s immunity (23). Arginine, an essential amino acid in poultry, is extensively involved in protein synthesis and possesses immune-regulatory and antioxidant functions (24). Cysteine contributes to sulfur-containing flavors in meat; it reacts with reducing sugars to form important sulfur-containing flavor compounds that generate meaty aromas upon heating (25). These SDMs were enriched in pathways related to amino acid metabolism, potentially influencing the formation of flavor amino acids through histidine metabolism; glycine, serine, and threonine metabolism; lysine degradation; cysteine and methionine metabolism; and tryptophan metabolism pathways. Tryptophan can be converted to serine via the tryptophan metabolism pathway. Serine then enters glycine, serine, and threonine metabolic pathways and is used to synthesize cysteine via cysteine biosynthesis. Cysteine participates in glutathione metabolism and is converted via glutathione into glutamate, which can subsequently be synthesized into glutamine. Histidine metabolism was a significantly enriched pathway in this study, with anserine and histamine identified within it. The abundance of histamine in Tengchong Snow chickens was lower than in AA chickens. Histamine is formed by the decarboxylation of histidine and can affect membrane permeability. Anserine is a bioactive endogenous compound with strong buffering, antioxidant, and vasodilatory effects and is considered a beneficial nutrient in meat (26). Chicken rich in anserine may be useful in managing hyperacidemia, gout, and Alzheimer’s disease (27–29).

Nucleotides are also important taste substances, but they o exhibit umami characteristics only when linked to a 5′ carbon atom. The freshness in nucleotides is mainly provided by IMP, GMP, and AMP, which can also act synergistically with L-glutamate to enhance the savory flavor of meat (30). In this study, the abundances of GMP, UMP, CMP, adenosine, and inosine in Tengchong Snow chickens were higher than that in AA broilers. Adenosine and inosine are involved in the synthesis of AMP and IMP, respectively. These SDMs were enriched in purine metabolism and pyrimidine metabolism pathways. Purine metabolism is a significant metabolic pathway. In this pathway, adenosine is one of the raw materials for synthesizing AMP, which can be used to synthesize AMP. AMP generates IMP under the action of adenine deaminase, GMP generates IMP under the action of guanine deaminase, and IMP is converted into inosine through dephosphorylation. UMP is formed by the action of cytosine deaminase in the pyrimidine metabolism pathway. The abundance of Lipoyl-AMP is upregulated, and the production of AMP is accompanied by activated Lipoyl-AMP partial transfer (31). Furthermore, the abundances of nicotinamide mononucleotide (NMN) and NAD were higher in Tengchong Snow chickens than in AA broilers. These compounds were enriched in nicotinate and nicotinamide metabolic pathways. NMN is a nucleotide with significant biological activity, widely present in various organisms. It acts as a coenzyme for certain dehydrogenases that catalyze redox reactions, enhances oxidative metabolism in skeletal muscle mitochondria, improves energy supply, and plays beneficial roles in delaying aging, treating diabetes, and managing neuropathic diseases (32). NMN is a precursor of NAD+, and its supplementation could rapidly elevate the level of NAD + in the body (33). NAD is a key coenzyme linking the respiratory chain and the tricarboxylic acid cycle. It serves as an electron carrier, transferring H + to flavoproteins, and participates in energy metabolism processes such as gluconeogenesis, glycolysis, and the tricarboxylic acid cycle. Within mitochondria, NAD + contributes to ATP generation through the tricarboxylic acid cycle and can be degraded to form AMP (34). AMP, as an important umami nucleotide, plays a significant role in the flavor formation. Both Lipoyl-AMP and NAD can produce AMP through a series of biochemical reactions, which may have a certain influence on meat flavor.

The abundances of pantothenic acid and arachidonic acid in Tengchong Snow chickens were higher than that in AA broilers, while tridecanoic acid was less abundant. Tridecanoic acid is a saturated fatty acid, and excessive intake could raise total cholesterol levels and is thus considered unfavorable for health (35). Arachidonic acid is an unsaturated fatty acid and an important precursor of meat flavor. Its products, such as hexanal, 1-octene-3-ol and 2-amylfuran, directly affect the composition of volatile flavor components and thus change the flavor of meat (36). Fatty acid biosynthesis and unsaturated fatty acid biosynthesis both utilize acetyl-CoA as a substrate and participate in fatty acid synthesis via the tricarboxylic acid cycle. Studies suggest that pantothenic acid acts as a potential flavor precursor (37). Pantothenic acid supports the synthesis of coenzyme A and acyl carrier proteins, through which it participates in metabolic activities. It plays important roles in fatty acid synthesis and degradation, acetyl and acyl group transfer, and various other anabolic and catabolic processes in the body (38). In summary, the SDMs mentioned above may represent key metabolites contributing to the superior flavor of Tengchong Snow chickens, with histidine and purine metabolism pathways likely serving as important routes affecting the formation of flavor precursors in this breed.

Through transcriptome analysis, 146 upregulated genes and 415 downregulated genes were identified. KEGG enrichment analysis of these DEGs revealed 12 significant enrichment pathways, which are all related to amino acid metabolism and may play an important role in the formation of flavor amino acids. By comparing the KEGG pathways enriched by 42 SDMs and 561 DEGs, it was found that there were 1 shared metabolic pathways: histidine metabolism. Histidine metabolism directly affects meat quality through its derivatives carnosine and histamine (39). Carnosine (*β*-alanyl-histidine) in muscle is an important antioxidant and pH buffer. It protects the integrity of muscle proteins and cell membranes by directly scavenging reactive oxygen species, inhibiting lipid peroxidation, and suppressing the formation of advanced glycation end products (40). Under stress or spoilage conditions, histidine is decarboxylated to form histamine, which activates pro-inflammatory signaling through receptors such as H1R. This can exacerbate pre-slaughter stress responses, affect glycogen metabolism, and ultimately lead to a decline in meat quality. Therefore, the balance of histidine metabolism is a key biochemical signaling node determining meat quality and stability.

Spearman analysis was performed on flavor related metabolites and differentially expressed genes in the KEGG enrichment pathway, identifying differentially expressed genes significantly associated with flavor precursor substances. *ASNS* showed a strong correlation with glutamine. Although widely present in mammalian organs, the function of *ASNS* in poultry remains less studied; it is known to influence muscle synthesis (41). The protein encoded by *ASNS* relies on glutamine to synthesize asparagine in ATP-dependent reaction (42). *CARNS1*, an ATP-dependent enzyme, is primarily involved in carnosine synthesis and modulates L-glutamine, L-histidine, and carnosine within the histidine metabolic pathway. It also participates in the metabolism of arginine, proline, *β*-alanine, and other compounds (43). Results indicated that *CARNS1* was highly correlated with glutamine, glutamate, and arginine. *CTH* not only catalyzes the breakdown of cystathionine to cysteine, but also converts cysteine into pyruvate, ammonia, and H₂S, playing a key role in the cysteine sulfur-transfer pathway (44). Analysis revealed a significant correlation between *CTH* and cysteine. Glutamine and glutamate also showed relatively high correlation coefficients with *GADL1*. *GADL1* encodes an acidic amino acid decarboxylase associated with umami-related amino acids; it influences chicken flavor by catalyzing the decarboxylation of aspartic acid and cysteine into flavor amino acids such as alanine (45). Study showed that *GADL1* knockout occurs β-Deficiency of alanine, carnosine, and goose carnosine (46). It is thus speculated that *ASNS*, *CARNS1*, *CTH*, and *GADL1* may be key genes regulating flavor amino acids in Tengchong Snow chicken muscle. *NME2* was significantly correlated with GMP, UMP, and CMP. The diphosphate kinase encoded by *NME2* converts triphosphate nucleotides into diphosphate nucleotides, which are further degraded to monophosphate nucleotides. *AMPD1* catalyzes the deamination of adenosine monophosphate to inosine monophosphate in skeletal muscle (47). Correlation analysis showed that *AMPD1* was highly correlated with GMP, which is converted to IMP via guanine nucleotide reductase, and *AMPD1* plays an important role in IMP interconversion. Previous studies have found that *AMPD1* could regulate the IMP content in chicken by participating in purine metabolism, thereby affecting its deposition (17). It was hypothesized that *NME2* and *AMPD1* are key genes regulating the formation of nucleotide-derived flavor substances. The genes selected in this study were based on strong correlations, and the exact causal regulatory mechanism remains to be verified through subsequent experiments such as gene knockout/ overexpression.

## 5 Conclusion

In summary, this study integrated metabolomics and transcriptomics analysis of the breast muscle tissues of Tengchong Snow chickens and AA broilers, identified histidine metabolism, cysteine and methionine metabolism, arginine and proline metabolism, purine metabolism pathway were key regulatory pathways for the formation of flavor precursors in Tengchong Snow chickens, and SDMs such as (5-L-Glutamyl)-L-glutamate, gamma-Glutamylalanine, S-Adenosylhomocysteine, Homo-L-arginine and GMP were key flavor precursors. Based on correlation analysis, it was speculated that *NME2*, *AMPD1*, *GADL1*, *ASNS*, *CARNS1*, and *CTH* were key genes regulating these SDMs.

## Data Availability

The data presented in the study are deposited in the NCBI Sequence Read Archive (SRA) repository, accession number PRJNA1427102.

## Footnotes

1
www.metaboanalyst.ca

2
http://ccb.jhu.edu/software/hisat2/index.shtml

3
https://github.com/mikelove/DESeq2

4
http://bioinfo.org/kobas/genelist/

5
https://www.originlab.com/
