# Supplementary material for: Integrated analysis of transcriptome and metabolome reveals the molecular interactions and regulation of muscle flavor precursors in Tengchong Snow chickens and AA broilers
Source: Front Vet Sci. 2026 Mar 10;13:1760840. doi: 10.3389/fvets.2026.1760840 (PMC13008670; doi:10.3389/fvets.2026.1760840)
Supplement: Supplementary file 1 [file Table_1.docx]

Table S1 Primer information of DEGs

| Genes | Gene accession number | Sequence of Primers (5’->3’) | Production length (bp) |
| --- | --- | --- | --- |
| ASNS | NM_001030977.2 | F: GTTTTGGTTTCCACCGCCTC | 137 |
|  |  | R: TCCAAACTGCTCCTGCAACT |  |
| CARNS1 | NM_001172593.1 | F: CCACCTAGTCGAAGGCAAGG | 102 |
|  |  | R: AGGTGGATCCGCAGGC |  |
| GADL1 | XM_046937979.1 | F: AAAGGGCTCTCAGGTTTGCC | 199 |
|  |  | R: GAAATGGTGCTGACCCCTCT |  |
| CTH | NM_205310.2 | F: CGTTGACGATGAGTACCCCG | 164 |
|  |  | R: TTATGAAGGGGTGGCCTGGG |  |
| AMPD1 | XM_040653018.2 | F: ATAGGGATCGGAGGCGTCA | 165 |
|  |  | R: CGAGTCCAACACGTCCCATT |  |
| NME2 | NM_205047.2 | F: CTCAACGTGGTGAAAACAGGC | 119 |
|  |  | R: CTGCCATGGATGATGTTTCTTCCC |  |
| β-actin | L08165.1 | F: GATATTGCTGCGCTCGTTGT | 127 |
|  |  | R: CAACCATCACACCCTGATGTC |  |

Table S2 Data quality and comparison results

| Sample | Raw Reads | Bases (bp) | Clean Reads | Clean Data (bp) | Q20 (%) | Q30 (%) | Mapped ratio(%) |
| --- | --- | --- | --- | --- | --- | --- | --- |
| AA1 | 44432576 | 6709318976 | 42205310 | 6373001810 | 98.2 | 94.84 | 80.45 |
| AA2 | 43901572 | 6629137372 | 41321366 | 6239526266 | 98.16 | 94.67 | 83.30 |
| AA3 | 40848356 | 6168101756 | 38471690 | 5809225190 | 98.15 | 94.65 | 84.39 |
| AA4 | 45892820 | 6929815820 | 43245432 | 6530060232 | 98.29 | 95.16 | 84.34 |
| AA5 | 45347528 | 6847476728 | 42677626 | 6444321526 | 98.17 | 94.79 | 84.87 |
| AA6 | 48600792 | 7338719592 | 45749582 | 6908186882 | 98.02 | 94.41 | 84.03 |
| TC1 | 45226704 | 6829232304 | 42715606 | 6450056506 | 97.97 | 94.34 | 85.13 |
| TC2 | 46074468 | 6957244668 | 43514032 | 6570618832 | 98.28 | 95.08 | 86.29 |
| TC3 | 38652038 | 5836457738 | 36258790 | 5475077290 | 97.97 | 94.42 | 84.24 |
| TC4 | 47563332 | 7182063132 | 44763540 | 6759294540 | 98.17 | 94.91 | 83.18 |
| TC5 | 51719226 | 7809603126 | 48386862 | 7306416162 | 98.01 | 94.49 | 83.97 |
| TC6 | 47587814 | 7185759914 | 44440578 | 6710527278 | 98.06 | 94.54 | 85.21 |
| Total | 545847226 | 153.44GB | 513750414 | 144.5GB |  |  |  |
